# Integration of GPCR Signaling and Sorting from Very Early Endosomes via Opposing APPL1 Mechanisms

**DOI:** 10.1016/j.celrep.2017.11.023

**Published:** 2017-12-05

**Authors:** Silvia Sposini, Frederic G. Jean-Alphonse, Mohammed A. Ayoub, Affiong Oqua, Camilla West, Stuart Lavery, Jan J. Brosens, Eric Reiter, Aylin C. Hanyaloglu

**Affiliations:** 1 Institute of Reproductive and Developmental Biology, Department of Surgery and Cancer, Imperial College London, London W12 0NN, UK; 2 Laboratory for GPCR Biology, Department of Pharmacology and Chemical Biology, University of Pittsburgh School of Medicine, Pittsburgh, PA 15261, USA; 3 PRC, INRA, CNRS, IFCE, Université de Tours, Nouzilly 37380, France; 4 Hammersmith Hospital, Imperial College NHS Trust, London W12 0NN, UK; 5 Division of Biomedical Sciences, Warwick Medical School, University of Warwick, Coventry CV4 7AL, UK; 6 Tommy’s National Miscarriage Research Centre, University Hospitals Coventry & Warwickshire, Coventry CV4 7AL, UK

**Keywords:** G-protein-coupled receptor, endosome, APPL1, recycling, cAMP

## Abstract

Endocytic trafficking is a critical mechanism for cells to decode complex signaling pathways, including those activated by G-protein-coupled receptors (GPCRs). Heterogeneity in the endosomal network enables GPCR activity to be spatially restricted between early endosomes (EEs) and the recently discovered endosomal compartment, the very early endosome (VEE). However, the molecular machinery driving GPCR activity from the VEE is unknown. Using luteinizing hormone receptor (LHR) as a prototype GPCR for this compartment, along with additional VEE-localized GPCRs, we identify a role for the adaptor protein APPL1 in rapid recycling and endosomal cAMP signaling without impacting the EE-localized β2-adrenergic receptor. LHR recycling is driven by receptor-mediated Gαs/cAMP signaling from the VEE and PKA-dependent phosphorylation of APPL1 at serine 410. Receptor/Gαs endosomal signaling is localized to microdomains of heterogeneous VEE populations and regulated by APPL1 phosphorylation. Our study uncovers a highly integrated inter-endosomal communication system enabling cells to tightly regulate spatially encoded signaling.

## Highlights

• GPCRs that internalize to very early endosomes (VEEs) require APPL1 to recycle
• Receptor recycling is driven by cAMP/PKA to phosphorylate serine 410 on APPL1
• cAMP signaling from GPCRs, such as LHR, occurs from distinct VEE microdomains
• APPL1 limits VEE cAMP signaling via opposing mechanisms required for GPCR sorting

## Graphical Abstract

**Figure:**
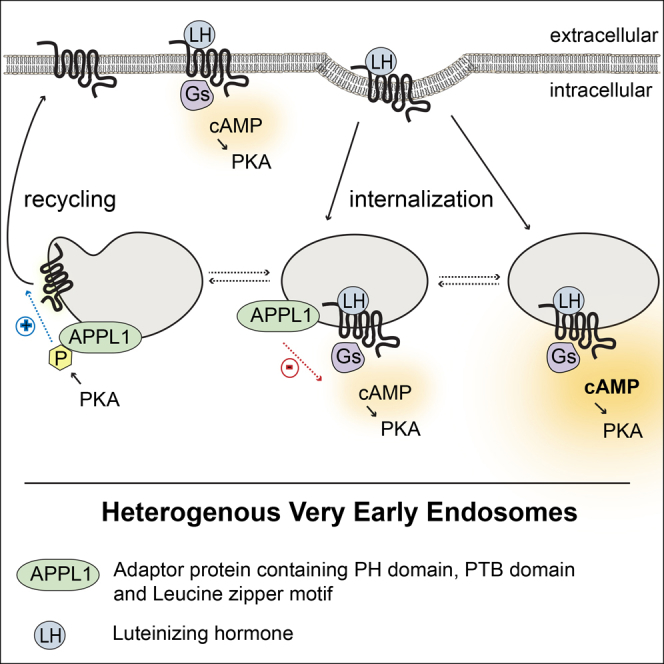

## Teaser

Sposini et al. report that G-protein signaling from specific GPCRs is spatially restricted in very early endosomes (VEEs), compartments distinct from classic early endosomes. Mechanistically, receptor signaling and sorting from VEEs occur via opposing phosphorylation states of APPL1 adaptor protein, providing novel decoding mechanisms for cells to tightly control signaling.

## Introduction

Within any cellular signaling system, the spatial organization of signaling networks is a critical mechanism for cells to decode complex pathways to specific downstream responses. Thus, membrane trafficking and signaling are viewed as an integrated system that mediates diverse fundamental cellular programs (Arora et al., 2007, Gonnord et al., 2012). Controlling signal location via membrane trafficking is highly relevant for the largest family of signaling receptors, the G-protein-coupled receptors (GPCRs). A well-studied role that trafficking plays in GPCR signaling is in the regulation of heterotrimeric G-protein signaling from the plasma membrane. A simple, but pertinent, example is the divergent sorting of GPCRs following ligand-induced endocytosis to recycling or degradative/lysosomal pathways, a process that is highly regulated and essentially produces opposite effects on cell-surface receptor signaling (Hanyaloglu and von Zastrow, 2008). Therefore, altering receptor trafficking profoundly reprograms GPCR signal transduction and physiologically represents a mechanism for cells to adapt to dynamic extracellular milieu. While under pathophysiological conditions, this can lead to perturbed GPCR signaling and disease (Sobolik et al., 2014, Barak et al., 2001). Recent studies, however, have demonstrated that G-protein signaling can continue, or be reactivated, following receptor internalization (reviewed in Sposini and Hanyaloglu, 2017, Irannejad et al., 2015), highlighting a key functional role of the endocytic system in GPCR activation. However, how membrane trafficking spatially decodes complex signaling pathways remains a fundamental outstanding biological question.

Following endocytosis, cell-surface receptors are trafficked to early endosomes (EEs) that are classically considered to be the primary sorting compartment for all internalized cargo (Hanyaloglu and von Zastrow, 2008, Goh and Sorkin, 2013). We have reported that GPCRs can be differentially sorted in the endosomal network. Based on analysis of the human luteinizing hormone receptor (LHR) and the β2-adrenergic receptor (B2AR), we demonstrated that the former receptor is targeted to very early endosomes (VEEs), a physically and biochemically distinct endosomal compartment from the classic EE to which the B2AR internalizes for its sorting (Jean-Alphonse et al., 2014). VEEs are smaller endosomes devoid of EE and intermediate EE markers such as EE antigen 1 (EEA1), Rab5, and phosphatidylinositol-3 phosphate (PI3P) (Jean-Alphonse et al., 2014). In contrast to EEs, a subpopulation of VEEs contain the multi-functional adaptor protein APPL1 (adaptor protein containing PH domain, PTB domain and leucine zipper motif) (Broussard et al., 2012, Cleasby et al., 2011, Lee et al., 2011, Jean-Alphonse et al., 2014), although its function in this compartment is unknown. We demonstrated that routing of LHR to the VEE is dependent on interactions with the PDZ domain containing protein Gαi-interacting protein C terminus (GIPC) via the LHR intracellular carboxy-terminal tail (C-tail) and that the sorting of LHR to VEEs is essential for its recycling back to the plasma membrane (Hirakawa et al., 2003, Jean-Alphonse et al., 2014). The VEE also represents a class of signaling endosome involved in sustained ERK1/2 activation in response to LHR signaling (Jean-Alphonse et al., 2014). Other GPCRs also traffic to the VEEs, such as the follicle-stimulating hormone receptor (FSHR) and the β1-adrenergic receptor (B1AR) (Jean-Alphonse et al., 2014). Thus, in addition to the compartmental bias in GPCR signaling between the plasma membrane and EEs (Lohse and Calebiro, 2013, Tsvetanova and von Zastrow, 2014), there is also compartmental bias across distinct endosomes, i.e., between the VEE and EE. These observations suggest that cells modulate GPCR signals by altering receptor sorting to different endosomal compartments. How spatially encoded signals are regulated within an endosomal system that comprises multiple and functionally heterogenic compartments remains poorly understood.

In this study, we demonstrate a central role for APPL1 in directing receptor sorting and endosomal G-protein signaling from the VEE. Furthermore, we provide evidence of functional heterogeneity within VEEs and an inter-endosomal communication system that enables cells to tightly regulate and reprogram dynamic GPCR signaling within the endocytic network.

## Results

### APPL1 Is Essential for GPCR Recycling via the VEE

**Figure 1:**
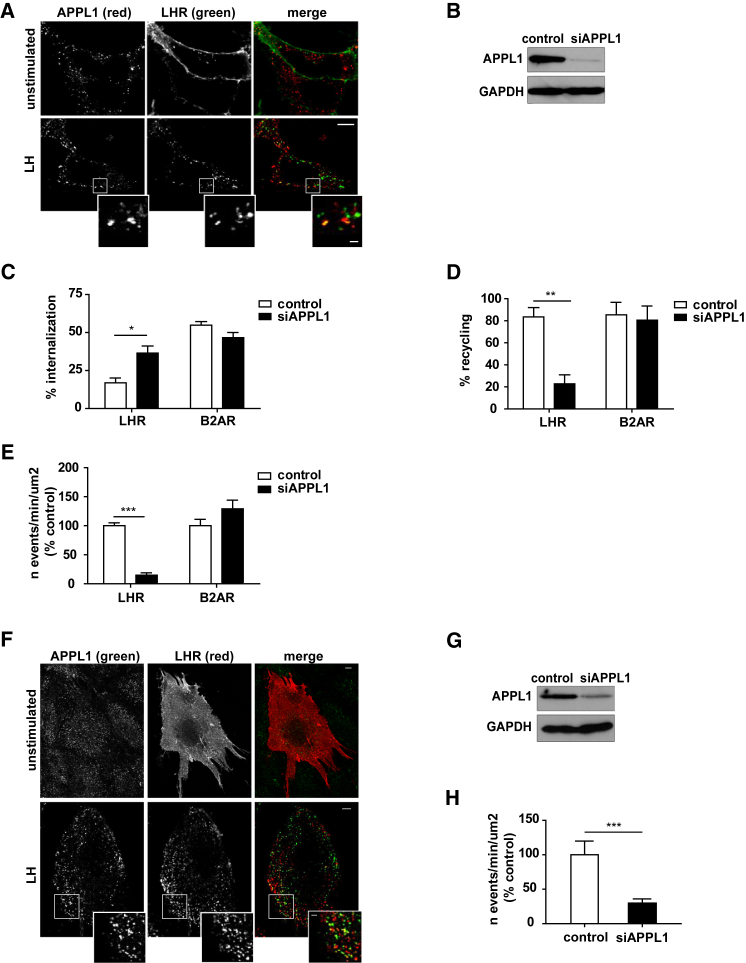
APPL1 Is Essential for LHR Recycling from VEEs (A) Confocal images of FLAG-LHR (green) and endogenous APPL1 (red) in cells with or without stimulation with LH (15 min). Scale bar, 5 μm; scale bar in inset, 1 μm. (B) Western blot of total cellular levels of APPL1 from cells treated with scramble or APPL1 siRNA. GAPDH was used as a loading control. (C and D) Flow cytometry analysis of cells expressing FLAG-LHR or FLAG-B2AR for ligand-induced internalization (15 min) (C) and recycling (1-hr ligand washout) (D) in cells treated with scramble or APPL1 siRNA. n = 4 independent experiments. ^∗^p < 0.05; ^∗∗^p < 0.01. (E) Recycling of SEP-LHR and SEP-B2AR was measured in real time, via TIR-FM, in cells treated with scramble or APPL1 siRNA 5 min after ligand addition. n = 16 cells per condition for LHR and 13 cells per condition for B2AR across at least 3 independent experiments. ^∗∗∗^p < 0.001. (F) Confocal images of FLAG-LHR (red) and endogenous APPL1 (green) in primary hESCs with or without stimulation with LH (15 min). Ligand-treated cells were “stripped” by PBS/EDTA (to remove surface-bound FLAG antibody). Scale bars, 5 μm; scale bar in inset, 1 μm. (G) Western blot of total cellular levels of APPL1 from hESC lysates following transfection with scramble or APPL1 siRNA. GAPDH was used as loading control. (H) SEP-LHR recycling in hESCs following siRNA-mediated knockdown of APPL1 was analyzed as in (E) . n = 29 cells per condition collected across 3 independent experiments. ^∗∗∗^p < 0.001. Data indicate mean ± SE. See also Figures S1–S3 and Movies S1 and S2.

Following ligand-dependent internalization, LHRs are sorted to VEEs, which are physically and biochemically distinct from EEs. Analysis of endogenous levels of the endosomal adaptor protein APPL1, demonstrated that this adaptor protein was localized to a subset of VEEs containing LHR (Figure 1A; Pearson’s correlation coefficient [PCC] = 0.337 ± 0.023 in unstimulated cells, and 0.786 ± 0.028 for LH-treated cells), consistent with our prior observations with GFP-tagged APPL1 (Jean-Alphonse et al., 2014). Therefore, to identify the functional impact of APPL1 on LHR endosomal organization and post-endocytic sorting, APPL1 was depleted using small interfering RNA (siRNA) in HEK293 cells stably expressing FLAG-tagged LHR (FLAG-LHR) (Figure 1B). LH-induced internalization and recycling of FLAG-LHR was quantitated by flow cytometry. APPL1 knockdown significantly increased the amount of receptor internalized (Figure 1C). This is likely to be due to the strong inhibition of LHR recycling of internalized receptor upon ligand washout (Figure 1D). Confocal microscopy confirmed that APPL1 knockdown prevented recycling of not only LHR but additional GPCRs previously shown to traffic to VEEs (Jean-Alphonse et al., 2014), the FSHR, and B1AR (Figure S1). To ascertain whether APPL1 is also essential for the recycling of GPCRs that are organized to the EE, but not the VEE, cells stably expressing FLAG-tagged B2AR were transfected with non-targeting or APPL1 siRNA. Quantitative analysis of isoproterenol-induced internalization and recycling by flow cytometry showed no differences in B2AR trafficking following APPL1 depletion (Figures 1C and 1D).

We next assessed the role of APPL1 in rapid GPCR recycling, using live-cell total internal reflection fluorescent microscopy (TIR-FM) and a pH-sensitive GFP super-ecliptic pHluorin (SEP) tagged at the extracellular N terminus of LHR. This GFP variant is highly fluorescent when located at the cell surface (neutral pH), yet its fluorescence is rapidly quenched in the lumen of endocytic vesicles (acidic pH), thus enabling analysis of dynamic GPCR recycling as it reinserts into the plasma membrane at single-event resolution (Miesenböck et al., 1998, Yudowski et al., 2007, Jullié et al., 2014). TIR-FM imaging of SEP-tagged LHR (SEP-LHR) revealed that recycling events appear as transient intense fluorescent spots upon reinsertion of the receptor in the plasma membrane (Figure S2A). These transient events at the cell surface have an average duration of 1.46 ± 0.12 s (Figure S2B) and are referred to as “puffs” (Yudowski et al., 2007). The appearance of puffs increased significantly within 5 min of LH treatment, with the number of events remaining constant over the imaging period (Figures S2C and S2D). The plasma membrane insertion events observed by TIR-FM were not affected by pre-treatment of cells with the protein synthesis inhibitor cyclohexamide (Figure S2E), indicating that receptor *de novo* synthesis does not contribute to recycling and consistent with our confocal imaging of receptor recycling that tracks the fate of the internalized receptor (Figure S1). Critically, recycling of SEP-LHR, but not SEP-tagged B2AR (SEP-B2AR), was strongly inhibited in APPL1-depleted cells (Figure 1E; Movies S1 [control] and S2 [APPL1 siRNA]), confirming that APPL1 specifically modulates VEE-sorted GPCRs. The inhibition of LHR recycling in APPL1-depleted cells was not a consequence of rerouting the receptor to the EE as APPL1 knockdown did not affect cellular levels of GIPC or the size of LHR endosomes (Figures S3A and S3B). We previously reported that VEEs are a third smaller in diameter compared to EEs (Jean-Alphonse et al., 2014). Co-localization of LHR with the EE marker EEA1 demonstrated a small but significant increase following knockdown of APPL1 (<10%); however, this increase was marginal compared to the 3-fold increase in EEA1 co-localization of LHR following GIPC knockdown (Figure S3C).

To examine whether APPL1-dependent recycling by LHR is conserved in cells that express LHR endogenously, we used primary human endometrial stromal cells (hESCs) (Bernardini et al., 2013). In hESCs, LHR also internalized from the plasma membrane to an endosomal compartment where a subpopulation was positive for endogenous APPL1 (32.33 ± 1.04%; PCC = 0.1233 ± 0.021 in unstimulated cells and 0.849 ± 0.027 in LH-treated cells; n = 15 cells; Figure 1F). Critically, rapid recycling of the receptor, assessed via TIR-FM, was APPL1 dependent (Figures 1G and 1H). Taken together, these data demonstrate that APPL1 has a specific role in sorting VEE-localized receptors to a recycling pathway. Moreover, the loss of APPL1-mediated LHR recycling leads to receptor accumulation in a population of VEEs.

### APPL1-Dependent Recycling Requires Protein Kinase A Phosphorylation at Serine 410

**Figure 2:**
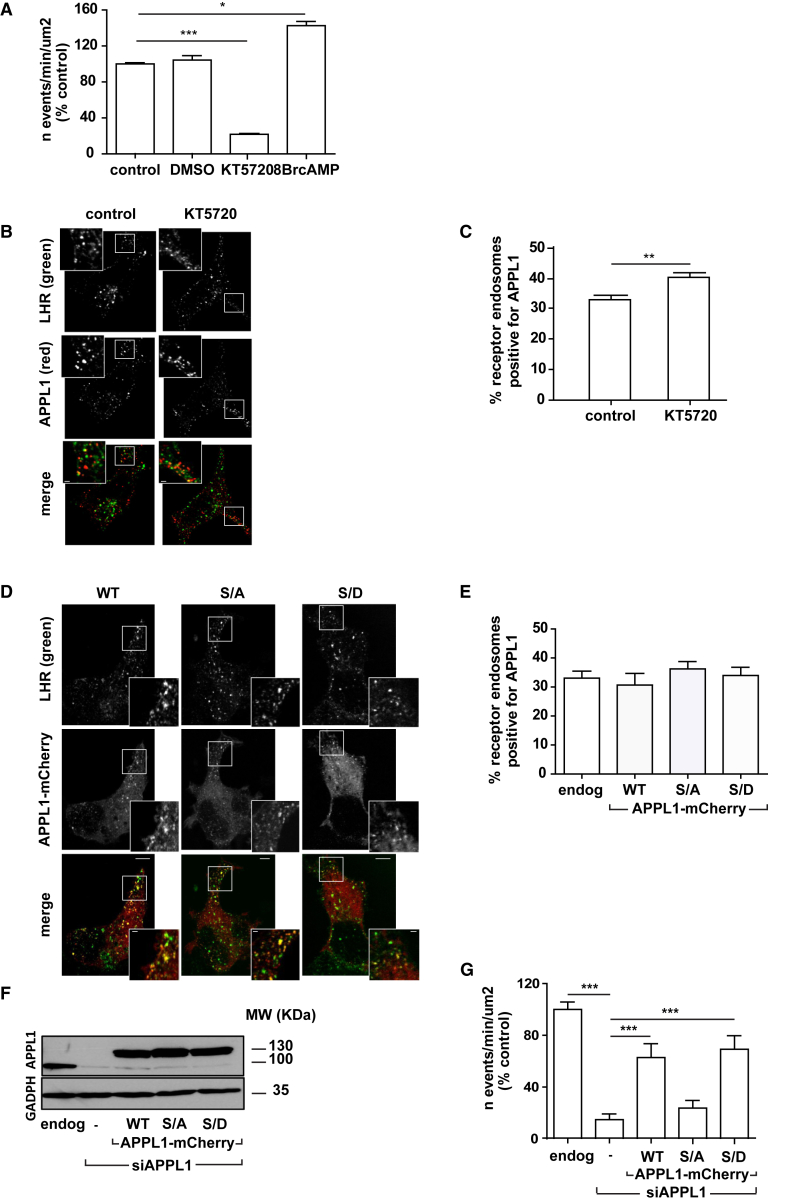
APPL1-Dependent Recycling of LHR Is Driven by cAMP/PKA Signaling and APPL1 S410 (A) SEP-LHR recycling was measured in real time by TIR-FM in the presence of LH in HEK293 cells pre-treated with either DMSO, PKA inhibitor KT5720 (10 μM, 15 min), or PKA activator 8-Br-cAMP (0.5 mM, 15 min). n = 16 cells per condition collected across 3 independent experiments. ^∗^p < 0.05; ^∗∗∗^p < 0.001. (B) Confocal images of FLAG-LHR (green) and endogenous APPL1 (red) in cells stimulated with LH (15 min) with or without KT5720 pre-treatment (10 μM, 15 min). Scale bars in insets, 1 μm. (C) Quantification of LHR endosomes positive for endogenous APPL1 from (B). n = 15 cells per condition, collected across 3 independent experiments. ^∗∗^p < 0.01. (D) Confocal images of FLAG-LHR (green) and either mCherry-WT, -S410A (S/A) or -S410D (S/D) APPL1 in cells stimulated with LH (15 min) Scale bars, 5 μm; scale bars in insets, 1 μm. (E) Quantification of (B) (endog) and (D) (WT, S/A, and S/D); n = 15 cells per condition, collected across 3 independent experiments. (F) Western blot analysis of total cellular levels of APPL1 from cells expressing SEP-LHR and transfected with mock (endog), siAPPL1 (-), siAPPL1 + mCherry-WT or mCherry-S410A (S/A), or mCherry-S410D (S/D) APPL1. GAPDH was used as loading control. (G) SEP-LHR recycling measured by TIR-FM in cells transfected as in (F). n ≥ 16 cells per condition imaged across at least 3 independent experiments. ^∗∗∗^p < 0.001. Data indicate mean ± SE.

We next examined the molecular mechanisms underpinning APPL1-dependent LHR recycling. GPCR activation and signaling are essential for subsequent intracellular trafficking of receptors (Vistein and Puthenveedu, 2013, Rosciglione et al., 2014). As LHR is primarily a Gαs-coupled receptor, a heterotrimeric G-protein pathway that activates adenylate cyclase and increases intracellular levels of the second messenger cyclic AMP (cAMP), we first assessed whether cAMP and its effector protein kinase A (PKA) regulate LHR recycling. SEP-LHR-expressing cells were pre-treated with a PKA inhibitor (KT5720) or activator (8-bromo-cAMP [8-Br-cAMP]) prior to stimulation with LH and live TIR-FM imaging. KT5720 potently inhibited LHR recycling compared to untreated or DMSO-treated cells (Figure 2A). By contrast, cells pre-treated with 8-Br-cAMP exhibited a significant increase in ligand-dependent LHR recycling (Figure 2A).

Given the absolute dependence of LHR recycling on APPL1, inhibition of PKA may impact recycling by altering trafficking of internalized LHR to APPL1 endosomes or by disrupting the endosomal localization of APPL1. The ability of APPL1 to localize to endosomes was unperturbed following treatment with KT5720 (Figure 2B; Table S1); however, the number of LHR endosomes positive for endogenous APPL1 increased significantly (Figures 2B and 2C). Thus, inhibition of PKA does not impair LHR sorting to APPL1 endosomes, suggesting that the loss of recycling under these conditions leads to receptor retention within this endosomal population.

PKA positively or negatively regulates recycling of other GPCRs via phosphorylation of receptors, or associated adaptor proteins (Man et al., 2007, Nooh et al., 2014). The human LHR does not contain PKA consensus phosphorylation sites in its intracellular domains (prediction conducted using the NetPhos 3.1 server) (Blom et al., 2004). Although APPL1 is phosphorylated on distinct sites by different putative kinases (Gant-Branum et al., 2010), serine 410 (S410) has been demonstrated to be phosphorylated by PKA (Erdmann et al., 2007). To determine whether phosphorylation of APPL1 on S410 mediates APPL1-dependent recycling, both a phospho-deficient mutant (S410A) and a phospho-mimetic mutant (S410D) were used. To exclude the possibility that the mutations disrupt the endosomal co-localization between LHR and APPL1, we first measured the percentage of LHR-containing endosomes positive for either endogenous APPL1 (Figures 2D and 2E) or mCherry-tagged wild-type (WT), S410A (S/A), or S410D (S/D) APPL1 mutants after LH stimulation. Co-localization of mCherry-tagged WT, S/A, or S/D APPL1 with LHR was comparable to the level observed with endogenous APPL1 (Figures 2D and 2E; PCC values in Table S1). Both WT and mutant mCherry-APPL1 were expressed at equivalent levels following siRNA-mediated depletion of endogenous APPL1 (Figure 2F). The loss of LHR recycling in cells depleted of endogenous APPL1 was restored upon expression of either WT or the phospho-mimetic mutant, S/D (Figure 2G). Notably, expression of the phospho-deficient S/A APPL1 mutant did not rescue LHR recycling (Figure 2G).

**Figure 3:**
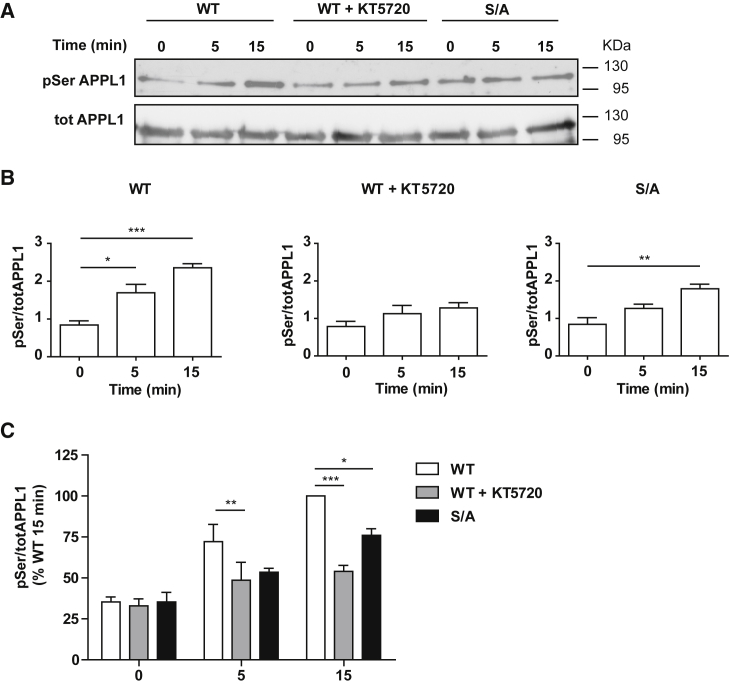
LHR Activation Induces PKA-Dependent Phosphorylation of APPL1, which Includes S410 Cells expressing FLAG-LHR were transfected with either WT or S/A GFP-APPL1 with or without stimulation with LH (5 and 15 min). Cells expressing WT GFP-APPL1 were also pre-treated with KT5720 (10 μM, 15 min). After collection of lysates, GFP-APPL1 was immunoprecipitated, and both phosphoserine and APPL1 levels were determined by western blot. (A) Representative immunoblot of phosphoserine (pSer) and total APPL1 (totAPPL1). (B) Densitometry analysis of APPL1 serine phosphorylation levels normalized to total APPL1. (C) Data are expressed as percentage of maximal response quantified (WT, 15 min LH). n = 3 independent experiments. ^∗^p < 0.05; ^∗∗^p < 0.01; ^∗∗∗^p < 0.001. Data indicate mean ± SE. See also Figure S4.

The aforementioned data indicate that APPL1-dependent recycling of LHR is driven by PKA-dependent phosphorylation of APPL1 at S410. To ascertain whether LH stimulation induces phosphorylation of APPL1 in a PKA-dependent manner, and specifically on S410, cells expressing WT APPL1-GFP were treated with LH and immunoprecipitated using a GFP nanobody. Eluates were analyzed by western blot using a phospho-serine antibody and an APPL1 antibody. Immunoprecipitation of APPL1 was only detected in cells transfected with APPL1-GFP (Figure S4A). The phospho-serine antibody detected a single band of ≈110 kDa, corresponding to APPL1-GFP, which increased following LH treatment (Figures 3A and 3B). Strikingly, the LH-dependent increase in phospho-serine levels of APPL1 was significantly inhibited by pre-treatment with KT5720 at both time points analyzed (Figures 3A and 3B). S/A APPL1 shows an increase in phosphorylation only after 15 min of LH stimulation, but this is significantly smaller than that induced on WT-APPL1 at the same time point. This suggests that APPL1 may be phosphorylated by PKA on other sites in addition to S410 (Figures 3A–3C). Stimulation of LHR-expressing cells with the PKA activator 8-Br-cAMP had no effect on APPL1 phosphorylation levels, suggesting that LH/LHR activation is required (Figure S4B). Furthermore, activation of the EE-localized GPCR, the B2AR, also did not increase levels of APPL1 phosphorylation (Figure S4C). Overall, these data suggest that LHR activation of the cAMP/PKA pathway drives its own recycling from VEEs via a mechanism that depends on ligand-dependent phosphorylation of APPL1 at PKA sites that include S410.

### APPL1 Negatively Regulates LH-Induced cAMP Signaling

**Figure 4:**
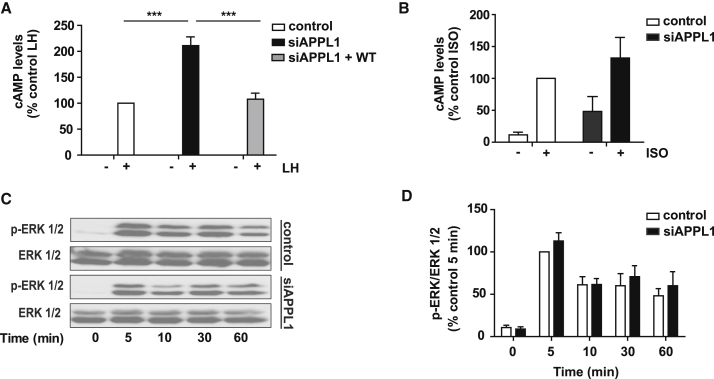
APPL1 Negatively Regulates LH-Induced cAMP Production (A) Intracellular levels of cAMP measured in cells stably expressing FLAG-LHR following transfection with either scramble (control), APPL1 siRNA (siAPPL1), or APPL1 siRNA and mCherry-APPL1 WT (siAPPL1 + WT). Cells were either not stimulated or stimulated with LH (5 min). n = 3 independent experiments. ^∗∗∗^p < 0.001. (B) Intracellular levels of cAMP were measured in cells stably expressing FLAG-B2AR following transfection with scramble or APPL1 siRNA and with or without stimulation with isoproterenol, (ISO; 5 min). n = 4 independent experiments. (C) Phosphorylation of ERK 1/2 was determined by western blot at stated time points after LH stimulation in FLAG-LHR cells treated with scramble or APPL1 siRNA. Total ERK was used as a loading control. (D) Densitometry analysis of ERK 1/2 phosphorylation was normalized to the 5-min control stimulation. n = 4 independent experiments. Data indicate mean ± SE. See also Figures S5 and S6.

Given that APPL1-dependent LHR recycling requires cAMP/PKA activation, we determined how APPL1 depletion reciprocally impacts LHR-mediated cAMP signaling. Agonist-induced cAMP production was measured in cells stably expressing FLAG-LHR and transfected with either non-targeting or APPL1 siRNA. There was no effect of APPL1 knockdown on the basal levels of cAMP; however, there was an unexpected increase in LH-induced cAMP levels following APPL1 knockdown (Figure 4A). This increase in cAMP signaling was not due to altered surface expression of LHR (LHR surface levels in cells treated with APPL1 siRNA, 102.20 ± 21.46% compared to cells treated with non-targeting siRNA; n = 4 independent experiments, p = 0.921) and was reversed upon transfection of WT APPL1 (Figure 4A). Furthermore, APPL1 depletion had a similar effect on the VEE-targeted B1AR and FSHR (Figures S5A and S5B) but did not impact ligand-induced cAMP signaling from the EE-targeted B2AR (Figure 4B). Depletion of endogenous APPL1 traps LHR primarily in the VEE (Figure S3), an endosomal compartment linked to sustained ERK signaling (Jean-Alphonse et al., 2014). However, APPL1 knockdown did not significantly alter LH-dependent ERK1/2 activation (Figures 4C and 4D). Collectively, these results demonstrate that APPL1 has a specific role in negatively regulating LHR-mediated cAMP but not ERK1/2 signal transduction.

### The Phosphorylation Status of APPL1 Regulates LHR-Dependent cAMP Signaling

**Figure 5:**
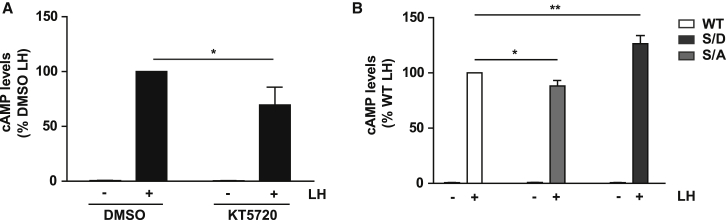
Regulation of cAMP Signaling via the Phosphorylation Status of APPL1 (A) Intracellular levels of cAMP measured in cells expressing FLAG-LHR following stimulation with LH (5 min) following pre-treatment with either DMSO or KT5720 (10 μM, 15 min); n = 3. ^∗^p < 0.05. (B) Intracellular levels of cAMP measured in FLAG-LHR cells following transfection with mCherry-WT (WT), S410A (S/A), or S410D (S/D) APPL1 and with or without stimulation with LH (5 min). n = 4 independent experiments. ^∗^p < 0.05; ^∗∗^p < 0.01. Data indicate mean ± SE.

APPL1 is essential for LHR recycling from the VEE via a mechanism that involves cAMP/PKA activation and phosphorylation of APPL1. In turn, APPL1 negatively regulates LHR signaling. Therefore, we examined whether PKA activation and APPL1 phosphorylation alter cAMP signaling. LHR-expressing cells pretreated with the PKA inhibitor KT5720 showed a partial but significant reduction in the levels of LH-stimulated cAMP production when compared to DMSO-treated cells (Figure 5A), conditions that increase the number of LHR endosomes in APPL1-positive VEEs (Figure 2) and inhibit LH-dependent APPL1 phosphorylation (Figure 3). This suggests that PKA activity may be a key determinant in the ability of APPL1 to negatively regulate LHR-induced cAMP signaling, independent of its role in receptor recycling. Therefore, we determined whether the phosphorylation status of APPL1 regulates LHR-dependent cAMP signaling from VEEs using cells expressing either WT, phospho-mimetic, or phospho-deficient APPL1. LH-induced increases in cAMP were not significantly different between cells transfected with or without WT APPL1 (WT APPL1: 110.50 ± 6.65% compared to untransfected cells, p = 0.165). A small but significant decrease in cAMP was observed in cells expressing the phospho-deficient S/A APPL1 compared to cells expressing WT APPL1, similar to the effect on LH-induced cAMP following inhibition treatment with KT5720 (Figures 5A and 5B). On the contrary, in cells expressing phospho-mimetic S/D APPL1, LH-induced cAMP was increased by ∼30% (Figure 5B). Overall, these data suggest that, when APPL1 is not phosphorylated on S410 by PKA, this has a repressive effect on cAMP production from LHR and that this repressive action of APPL1 is reversed when S410 is phosphorylated.

### LHR-Mediated cAMP Signaling from the VEE

**Figure 6:**
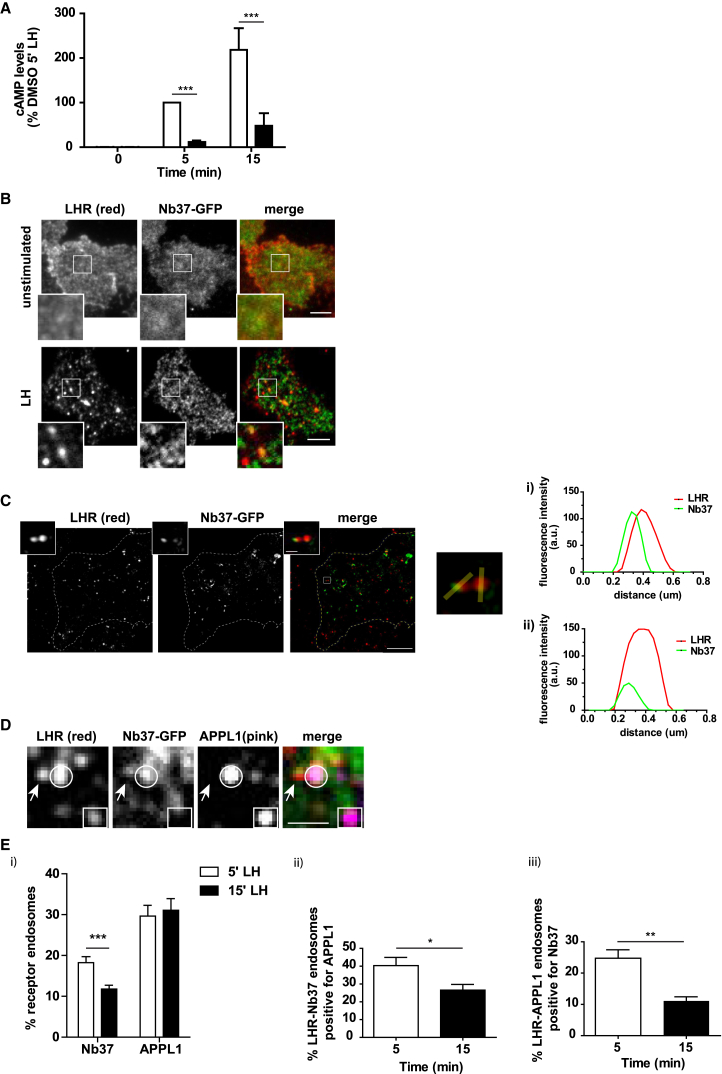
LHR Activates Gαs/cAMP Signaling from VEEs Heterogenous for APPL1 (A) Intracellular levels of cAMP measured in cells expressing FLAG-LHR with or without stimulation with LH (5 and 15 min) and pre-treatment with either DMSO or Dyngo-4a (30 μM, 45 min). n = 4 independent experiments. ^∗∗∗^p < 0.001. Data are expressed as cAMP levels normalized to 5 min of LH treatment (DMSO). (B) TIR-FM images of FLAG-LHR (red) and Nb37-GFP with or without stimulation with LH (15 min). Scale bars, 5 μm. (C) SIM images of FLAG-LHR (red) and Nb37-GFP following stimulation with LH. Scale bar, 5 μm. Inset shows the microdomain organization of Nb37 within individual LHR endosomes (scale bar, 500 nm). Line intensity analysis is shown for two endosomes (i and ii). Representative images are from n = 30 endosomes. (D) TIR-FM images of FLAG-LHR (red), Nb37-GFP (green), and endogenous APPL1 (pink) in cells stimulated with LH (5 or 15 min). Arrows indicate LHR endosome positive for Nb37 only; circles indicate LHR endosome positive for Nb37 and APPL1; squares indicate LHR endosome positive for APPL1 only. Scale bar, 1 μm. (E) Quantification of LHR endosomes positive for either APPL1 or Nb37 (i), LHR-Nb37 endosomes positive for APPL1 (ii), and LHR-APPL1 endosomes positive for Nb37 (iii) after 5 or 15 min of LH stimulation. n = 15 cells per condition from Figure 5D that were quantitated across 3 independent experiments. ^∗^p < 0.05; ^∗∗^p < 0.01; ^∗∗∗^p < 0.001. Data indicate mean ± SE.

The ability of APPL1 to negatively regulate cAMP levels of VEE-targeted GPCRs, yet positively regulate recycling via PKA, suggests that cAMP signaling occurs from endomembranes. To test this hypothesis, we assessed the proportion of LHR-mediated cAMP generated from intracellular compartments in cells treated with the inhibitor of the large GTPase, dynamin, a protein key in vesicle scission, including clathrin-coated vesicles, from the plasma membrane. Dyngo-4a is a potent dynamin inhibitor that blocks internalization of multiple GPCRs, including LHR and B2AR (Figure S6A) (McCluskey et al., 2013, Jean-Alphonse et al., 2014, Bowman et al., 2016). LH-induced cAMP signaling was strongly inhibited by Dyngo-4a pre-treatment following both acute and sustained LH treatment (percent inhibition compared to DMSO-treated cells is 88.02 ± 3.27% at 5 min and 79.86 ± 6.93% at 15 min of LH stimulation) (Figure 6A). Dyngo-4a did not significantly attenuate isoproterenol-induced intracellular cAMP production in B2AR-expressing cells (Figure S6B), which signals primarily, but not exclusively, at the plasma membrane (Irannejad et al., 2013, Tsvetanova et al., 2016). To spatially capture the subcellular location of Gαs activation, we used GFP-tagged nanobody 37 (Nb37-GFP), a biosensor that captures the activated, but nucleotide-free, state of Gαs (Irannejad et al., 2013). TIR-FM was used, as LHR/Nb37 endosomes were more prevalent in the peripheral region of cells, consistent with the peripheral distribution of APPL1 (Erdmann et al., 2007, Miaczynska et al., 2004). FLAG-LHR-expressing cells transfected with Nb37-GFP demonstrated a redistribution of Nb37 to a proportion of LHR endosomes (Figure 6B; PCC = 0.191 ± 0.042 in unstimulated cells and 0.771 ± 0.027 in LH-stimulated cells). GPCR endosomal signaling may be localized within endosomal microdomains (Varandas et al., 2016, Bowman et al., 2016). Due to the restrictive size of VEEs (∼400 nm), and because the diffraction limit of visible light is ∼200 nm (Abbe, 1873), we used structured illumination microscopy (SIM) (Gustafsson, 2000) on fixed cells. As shown in Figure 6C, a subpopulation of FLAG-LHR endosomes also contained Nb37-GFP. Due to the increase in both lateral resolution and axial resolution that SIM affords, we observed that Nb37 is not uniformly distributed within LHR endosomes but localizes to a sub-domain (Figures 6Ci and 6Cii).

APPL1-positive LHR endosomes represent almost half of the total receptor-occupied VEE, when accounting that ∼70% of LHR endosomes are not EEA1 positive (Figures 2C and S3C). To determine whether LHR endosomes exhibiting active Gαs co-localize with endogenous APPL1, 3-color TIR-FM analysis was used and revealed that LHR-Nb37 signaling endosomes were heterogeneous and characterized by LHR-Nb37 endosomes with and without APPL1, including LHR/APPL1 endosomes with no Nb37 (Figure 6D). Analysis of LHR-Nb37 endosomes after LH stimulation (5 and 15 min) revealed that ∼40% of LHR-Nb37 endosomes were marked by APPL1 after 5 min of LH stimulation and that this number decreased to 26% after 15 min (Figures 6Ei and 6Eii). Interestingly, although the number of LHR-Nb37 endosomes significantly decreased with time, the total number of LHR-APPL1 endosomes remained constant (Figure 6Ei). Consequently, the proportion of LHR-APPL1 endosomes with Nb37 significantly decreased over time (Figure 6Eiii). These data suggest that endosomal Gαs is acutely activated by LHR and that Nb37 temporally localizes to a subpopulation of LHR endosomes that also exhibit APPL1 co-localization.

## Discussion

Endocytic trafficking of GPCRs is recognized as a primary mechanism that cells use not only to define the pattern of cell-surface G-protein signaling but also to generate additional signaling platforms at the endomembrane (West and Hanyaloglu, 2015). Our study reveals how GPCR signaling is deeply integrated with endocytic trafficking and demonstrates that the endomembrane system represents a complex and exquisitely regulated network capable of inter-endosomal communication.

We previously reported that certain GPCRs are sorted to the VEE, an endosomal compartment with physical and biochemical properties distinct from those of the classic EE or its intermediates. Despite the fact that APPL1 was identified as the only marker of the VEE, its role in LHR function at the VEE was unknown (Jean-Alphonse et al., 2014). APPL1 has been implicated in the trafficking and signaling of a variety of membrane receptors as an EE endocytic intermediate that associates with the GTPase Rab5 (Lin et al., 2006, Thomas et al., 2011); however, we have already demonstrated that LHR trafficking and signaling is Rab5 independent (Jean-Alphonse et al., 2014). Here, we demonstrate two functions for APPL1. First, APPL1 is essential for rapid LHR recycling from VEEs to the plasma membrane, a role that has not yet been ascribed to APPL1 for any membrane cargo. Second, APPL1 functions as a negative regulator of LHR-mediated cAMP production from VEEs.

The inhibition of recycling by depletion of APPL1 is not due to redirecting LHR from VEEs to EEs. The small increase of LHR to EEA1-positive EEs following cellular depletion of APPL1 is likely a consequence of receptor accumulation and sequestration in endosomes due to the loss of the APPL1 compartment and inhibition of receptor recycling. APPL1 has been recently shown to occupy a small subpopulation of sorting endosomes, and even tubular structures from these endosomes, but these correspond to the morphologically larger EEA1-positive EEs, which are distinct from the smaller VEEs, and the role of APPL1 in the post-endocytic sorting was not assessed (Kalaidzidis et al., 2015). We showed that APPL1 is required for recycling of distinct GPCRs that traffic to the VEE, although not the EE, as recycling of B2AR was APPL1 independent. This specific role in post-endocytic trafficking of VEE-localized receptors is likely to be a conserved mechanism, as APPL1-dependent LHR recycling was recapitulated in primary hESCs.

Despite the divergent endosomal sorting of LHR and B2AR and their differential requirement for APPL1, the recycling of both GPCRs is regulated via their activation of Gαs-cAMP-PKA pathway. Interestingly, cAMP/PKA activation exerts opposite functions by phosphorylating distinct targets for these two GPCRs. Sequence-dependent recycling of B2AR is negatively regulated via PKA-dependent phosphorylation of the receptor C-tail (Vistein and Puthenveedu, 2013, Yudowski et al., 2009). By contrast, LHR-dependent Gαs/cAMP/PKA activation drives, rather than inhibits, receptor recycling and is dependent on phosphorylation of APPL1 by PKA. Interestingly, the VEE-localized B1AR, which also undergoes APPL1-dependent recycling, requires cAMP/PKA signaling to drive its post-endocytic sorting (Nooh et al., 2014, Gardner et al., 2007). LH-dependent phosphorylation of APPL1 is PKA mediated and includes S410. Although additional PKA sites may be phosphorylated in APPL1 by LHR activation, critically, S410 is essential for LHR recycling. Overall, our study demonstrates how GPCR signaling promotes its own receptor sorting via modulation of the trafficking machinery.

Our data also demonstrate a strong bidirectional relationship between GPCR signaling and endocytic trafficking. Not only does LHR-mediated cAMP signaling drive recycling via phosphorylation of APPL1, but APPL1 also exerts negative feedback on ligand-induced cAMP signaling from LHR. APPL1 is a known adaptor of signaling molecules, propagating signaling pathways such as AKT, GSK3-β, p38, and AMPK (Xin et al., 2011, Ryu et al., 2014) rather than inhibiting signaling pathways as demonstrated in this study for cAMP/PKA. Interestingly, negative regulation of LHR-mediated cAMP signaling is dependent on the phosphorylation status of APPL1 but in an opposing manner to its role in regulating LHR recycling. This negative regulation is consistent with the decreased presence of active LHR/Gαs signaling endosomes that contain APPL1 over time, with an increased proportion of LHR/APPL1 endosomes without active Gαs. This suggests that there are at least two forms of APPL1 across different VEEs (or, perhaps, even across microdomains within an individual VEE) that mediate distinct LHR functions as the receptor traverses the VEE compartment: (1) the phosphorylated S410 form of APPL1 necessary for LHR recycling and (2) APPL1 not phosphorylated at S410 to limit LHR cAMP signaling. Although we demonstrate that the primary source of cAMP signaling from LHR is endosomal, as Dyngo-4a could not completely block LHR-mediated cAMP signaling, this may suggest that some plasma membrane signaling—and, thus, the source of active PKA that phosphorylates APPL1—could be both plasma membrane and endomembrane derived. The inactivation of endosomal G-protein signaling, prior to receptor sorting to a recycling pathway, has been shown to be a necessary step for other GPCRs (Feinstein et al., 2013, McGarvey et al., 2016); thus, the role of APPL1 in negative regulation of VEE signaling is consistent with this. As with these prior studies on G-protein signaling from EEs, the level at which G-protein signaling is deactivated is unknown and, for the VEE and APPL1, could include modulation of receptor/G-protein coupling, modulation of cAMP generation, and/or degradation via adenylate cyclases and phosphodiesterases, respectively. Given that we observe a decrease in active Gαs over time, this may suggest modulation at the level of the G protein. Furthermore, as LHR may phosphorylate APPL1 via PKA at, as yet, unknown sites in addition to S410, this suggests that there may be a range of phosphorylated forms of APPL1 with additional functions within the VEE, such as sustained ERK signaling (Jean-Alphonse et al., 2014).

There has been an increasing number of reports that GPCRs can continue, or reactivate, G-protein signaling from endosomes (Sposini and Hanyaloglu, 2017, Irannejad et al., 2015), although GPCR/G-protein signaling from endosomes distinct from the EE has not previously been demonstrated. Interestingly, sustained cAMP signaling from the mouse LHR in the ovary is purportedly important for maintaining meiotic arrest in oocytes (Lyga et al., 2016). However, unlike human LHRs, rodent LHRs poorly internalize and do not associate with GIPC or recycle (Hirakawa et al., 2003, Nakamura et al., 2000). Accumulating evidence has highlighted the role of arrestin in endosomal G-protein signaling via its simultaneous association with receptor and Gα protein, including Gβγ subunits (Jean-Alphonse et al., 2017). This is pertinent for those GPCRs exhibiting sustained arrestin associations via receptor C-tail Ser/Thr clusters, which co-traffic with arrestin to endosomes (Thomsen et al., 2016, Kumari et al., 2016). There are likely to be multiple modes of endosomal signaling, as GPCRs such as the B2AR and LHR do not contain these C-tail clusters and associate with arrestin at clathrin-coated pits, yet both exhibit endosomal G-protein activation (Irannejad et al., 2013, Jean-Alphonse et al., 2014). Collectively, these prior studies have led to the model where GPCR/G-protein signaling is categorized into 2 phases: plasma membrane signaling and endomembrane signaling (West and Hanyaloglu, 2015, Lohse and Calebiro, 2013). This study, however, demonstrates that LHR-mediated cAMP signaling in human cells occurs from distinct VEE subpopulations. Together, the emerging model indicates that GPCR activity in the VEEs is highly heterogeneous. This heterogeneity seems to function as a mechanism to spatially restrict the cAMP microenvironment to a VEE subpopulation and mediate APPL1 phosphorylation, which then enables receptor sorting to a rapid recycling pathway, illustrating potential inter-endosomal communication of GPCR activity within the VEE compartment.

It is increasingly apparent that endocytic trafficking of GPCRs is critical to resolve, or decode, complex cellular signaling at a spatial level. The role of the endosomal network and machinery in regulating GPCRs is only just emerging. However, our discovery that endosomal heterogeneity and inter-endosomal communication are essential for coordinating GPCR signaling and sorting will enable the construction of cellular models that integrates GPCR signaling across multiple endosomal compartments, models that could provide insight into complex disease and therapeutic strategies for this highly significant superfamily of receptors.

## Experimental Procedures

### Reagents

The antibodies used were: mouse anti-FLAG (M1, Sigma); rabbit anti-APPL1 (Cell Signaling Technology); rabbit anti-EEA1 (Cell Signaling Technology); mouse anti-GAPDH (Millipore); rabbit anti-p42/44 ERK and phospho-p42/44 ERK (Cell Signaling Technology); rabbit anti-phosphoserine (Millipore); goat anti-rabbit and anti-mouse AlexaFluor 488, 555, 568, and 647 (Thermo Fisher); and goat anti-rabbit and anti-mouse horseradish peroxidase (HRP) (Thermo Fisher Scientific). The inhibitors/activators used were: Dyngo-4a (Abcam) at 30 μM (45 min pre-treatment), KT5720 (Abcam) at 10 μM (15 min pre-treatment), and 8-Br-cAMP (Sigma) at 0.5 mM (15 min pre-treatment). LH and follicle-stimulating hormone (FSH) (A.F. Parlow, National Hormone and Peptide Program, Harbor-UCLA Medical Center) were used at 10 nM, and isoproterenol (ISO; Sigma) was used at 10 μM. All concentrations of ligands used (LH and FSH at 10 nM and isoproterenol at 10 μM) induce maximal cAMP responses from dose-response curves published previously (Bouvier et al., 1987, Gudermann et al., 1992, Alvarez et al., 1999).

### DNA Constructs and siRNA

FLAG-hLHR, FLAG-hB2AR, FLAG-hFSHR, and GFP-WT APPL1 have been previously described (Jean-Alphonse et al., 2014). Nb37-GFP and SEP-hB2AR were kind gifts from Mark von Zastrow (University of California, San Francisco [UCSF], USA). SEP-LHR was obtained as follows: SEP was subcloned from SEP-B2AR using AgeI and ligated into FLAG-LHR, containing an AgeI restriction site in the FLAG sequence created by site-directed mutagenesis (QuikChange, Stratagene) using oligos corresponding to GTGTGGTCTCCGATTACACCGGTGATGATGATAAGCGAGC.

FLAG-hB1AR was purchased from Addgene. mCherry-WT APPL1 was generated via subcloning mCherry into GFP-WT APPL1 using SalI and XhoI sites. mCherry-S410A APPL1 and -S410D APPL1 were generated by site-directed mutagenesis (QuikChange, Stratagene) using oligos corresponding to GCAGAGGCACGAGGCCCTGCGGCCAGCAGC and GCAGAGGCACGAGGACCTGCGGCCAGCAGC, respectively. siRNA-mediated knockdown of APPL1 was achieved by transfection of duplex RNA oligos (Life Technologies) corresponding to GACAAGGTCTTTACTAGGTGTATTT. Control cells were transfected with non-sense duplex RNA oligos (AATTCTCCGAACGTGTCACG).

### Cell Culture and Transfection

HEK293 cells (ATCC) were maintained in DMEM containing 10% FBS and penicillin/streptomycin (100 U/mL) at 37°C in 5% CO_2_. Primary hESC cultures were established from endometrial biopsies, taken randomly in the cycle, as previously described (Brosens et al., 1999). Proliferating hESC monolayers were maintained in DMEM/F12 supplemented with 10% dextran-coated charcoal (DCC), antibiotic/antimycotic (100 U/mL), and L-glutamine (200 mM) at 37°C in 5% CO_2_. Primary cultures were passaged no more than three times and allowed to grow to confluency prior to decidualization with 8-Br-cAMP (0.5 mM) and 17α-medroxyprogesterone acetate (MPA) (10^−6^ M) for 72 hr. For both HEK293 cells and hESCs, transient and stable transfections of DNA were performed with Lipofectamine 2000 (Life Technologies). Transfection of siRNA was performed using RNAiMAX (Life Technologies). For transient expression, cells were assayed 48 and 96 hr post-DNA and -siRNA transfection, respectively.

### Flow Cytometry

Flow cytometry was used to quantitate the internalization and recycling of receptors by measuring the levels of cell-surface FLAG-tagged receptors as described previously (Hanyaloglu et al., 2005). Briefly, cells were fed with mouse anti-FLAG antibody (15 min, 37°C) prior to treatment with agonist (10 and 30 min). For receptor recycling, ligand-treated cells were washed and incubated in media for 1 hr. All treatments were carried out in triplicate. Cells were lifted with trypsin, and the cell suspension was washed with PBS and incubated with goat anti-mouse Alexa Fluor 488 or 647. The fluorescence intensity of 10,000 cells was collected for each sample using a flow cytometer (BD FACSCalibur, Becton Dickinson). Both the mean fluorescence and percentage of cells gated were quantified. The percentage of receptor recycling was calculated from the proportion of internalized receptor (as indicated by a decrease of immunoreactive surface receptor with agonist compared to unstimulated cells) that was recovered at the cell surface.

### Confocal Imaging

Receptor imaging in live or fixed cells was monitored by “feeding” cells with Alexa-Fluor 488- or 555-conjugated FLAG antibodies (15 min, 37°C) in phenol-red-free DMEM prior to agonist treatment. Fixed cells were washed three times in PBS/0.04% EDTA to remove FLAG antibody bound to the remaining surface receptors. Cells were imaged using a TCS-SP5 confocal microscope (Leica) with a 63× 1.4 numerical aperture (NA) objective. Leica LAS AF image acquisition software was utilized. All subsequent raw-image files were analyzed using ImageJ or LAS AF Lite (Leica) to measure endosome diameter size or level of co-localization. Pearson’s colocalization coefficient was calculated for at least 3 regions of interest (ROIs) per cell using the ImageJ plugin JACoP.

### Immunoprecipitation

Immunoprecipitation of GFP-APPL1 constructs was conducted using GFP-Trap agarose beads (ChromoTek) as per the manufacturer’s protocol. HEK293 cells transfected with GFP-APPL1 constructs were washed with ice-cold PBS three times, collected, and homogenized with lysis buffer (0.5% NP40, 10 mM Tris-HCl [pH 7.5], 150 mM NaCl, 0.5 mM EDTA, protease and phosphatase inhibitors) for 30 min. Lysates were centrifuged, and the supernatant was incubated for 2 hr with GFP-Trap agarose beads. Beads were washed three times and resuspended in elution buffer (120 mM Tris-HCl [pH 6.8], 20% glycerol, 4% SDS, 10% β-mercaptoethanol, 0.04% bromophenol blue). Samples were separated on a 12% SDS-PAGE gel.

### Signaling Assays

For measurement of ERK activation by western blot, cells were treated and lysed as described previously (Jean-Alphonse et al., 2014). Measurement of whole-cell cAMP was carried out with the cAMP Dynamic 2 kit (Cisbio Bioassays) as per manufacturer’s instructions. Cells were ligand treated in the absence of phosphodiesterase inhibitors in triplicate, and experiments were repeated at least three times. All cAMP concentrations were corrected for protein levels.

### TIR-FM

Cells were imaged using a Elyra PS.1 AxioObserver Z1 motorized inverted microscope with a scientific complementary metal-oxide-semiconductor (sCMOS) or electron-multiplying charge-coupled device (EMCCD) camera and an alpha Plan-Apochromat 100×/1.46 Oil DIC M27 Elyra objective (Zeiss), with solid-state lasers of 488 nm, 561 nm, and/or 642 nm as light sources. For live-cell imaging, cells were imaged live for 1 min at a frame rate of 10 frames per second (fps) at 37°C in phenol-red-free Opti-MEM supplemented with HEPES (Life Technologies). ZEN Lite image acquisition software was utilized to collect time-lapse movies and analyzed as tiff stacks using the ImageJ plugin Time Series Analyzer. The number of recycling events counted was normalized by cell area. For fixed-cell imaging, cells were prepared as for confocal imaging.

### SIM

Cells were imaged using an Elyra PS.1 AxioObserver Z1 motorized inverted microscope with a EMCCD camera and Plan-Apochromat 63×/1.4 Oil DIC (differential interference contrast) M27 Elyra objective (Zeiss) with solid-state lasers of 488 nm and 561 nm as light sources. ZEN lite software was used for both acquisition of z stacks (5 phases and 3 rotations grating) and reconstruction. Quality of raw and reconstructed data was determined using the ImageJ plugin SIM check (Ball et al., 2015). Cells were prepared as for confocal imaging.

### Patient Selection and Endometrial Sampling

The study was approved by the National Health Service National Research Ethics-Hammersmith and Queen Charlotte’s and Chelsea Research Ethics Committee (1997/5065). Pre-menopausal women were recruited from the Infertility Clinic at Hammersmith Hospital, Imperial College London NHS Trust. Written informed consent was obtained from all participants in accordance with the guidelines in the Declaration of Helsinki 2000. Samples were obtained using a Wallach Endocell sampler (Wallach), starting from the uterine fundus and moving downward to the internal cervical ostium. In total, 8 endometrial biopsies were processed for primary cultures in this study. The average age (±SD) of the participants was 32.5 ± 4.4 years.

### Statistical Analysis

Data are given as means ± SE. Statistical significance was determined using GraphPad Prism v5 (GraphPad, La Jolla, CA, USA). An unpaired Student’s t test, one-way ANOVA followed by Dunnett post-test, or two-way ANOVA followed by Bonferroni post-test was used when comparing two groups, more than two groups, or at least two groups under multiple conditions, respectively. Differences were considered significant at p < 0.05.

## Author Contributions

S.S., F.G.J.-A., M.A.A., A.O., C.W., and A.C.H. performed experiments. S.L. and J.J.B. provided human endometrial biopsies. S.S., F.G.J.-A., M.A.A., E.R., S.L., J.J.B., and A.C.H. designed research, analyzed data, and wrote the paper.

## Data and Software Availability

The accession numbers for the flow cytometry data reported in this paper are FlowRepository: FR-FCM-ZYDA and FR-FCM-ZYDB.

## Supplemental Information

Document S1. Figures S1–S6 and Table S1

Movie S1. Recycling of SEP-LHR Measured via TIR-FM, Related to Figure 1E Representative TIR-FM movies of HEK 293 cells stably expressing SEP-LHR treated with scramble siRNA. Cells were stimulated with LH (10 nM) and imaged live by TIRFM for 1 minute at 10 fps. Movies are shown at 30 fps.

Movie S2. Recycling of SEP-LHR Measured via TIR-FM Requires APPL1, Related to Figure 1E Representative TIR-FM movies of HEK 293 cells stably expressing SEP-LHR treated with APPL1 siRNA. Cells were stimulated with LH (10 nM) and imaged live by TIRFM for 1 minute at 10 fps. Movies are shown at 30 fps.

Document S2. Article plus Supplemental Information
