# Supplementary material for: Integration of GPCR Signaling and Sorting from Very Early Endosomes via Opposing APPL1 Mechanisms
Source: Cell Rep. 2017 Dec 5;21(10):2855–67. doi: 10.1016/j.celrep.2017.11.023 (PMC5732320; doi:10.1016/j.celrep.2017.11.023)
Supplement: Document S1. Figures S1–S6 and Table S1 [file mmc1.pdf]

Cell Reports, Volume *21*

Supplemental Information

Integration of GPCR Signaling and Sorting from Very Early Endosomes via Opposing APPL1 Mechanisms

Silvia Sposini, Frederic G. Jean-Alphonse, Mohammed A. Ayoub, Affiong Oqua, Camilla West, Stuart Lavery, Jan J. Brosens, Eric Reiter, and Aylin C. Hanyaloglu

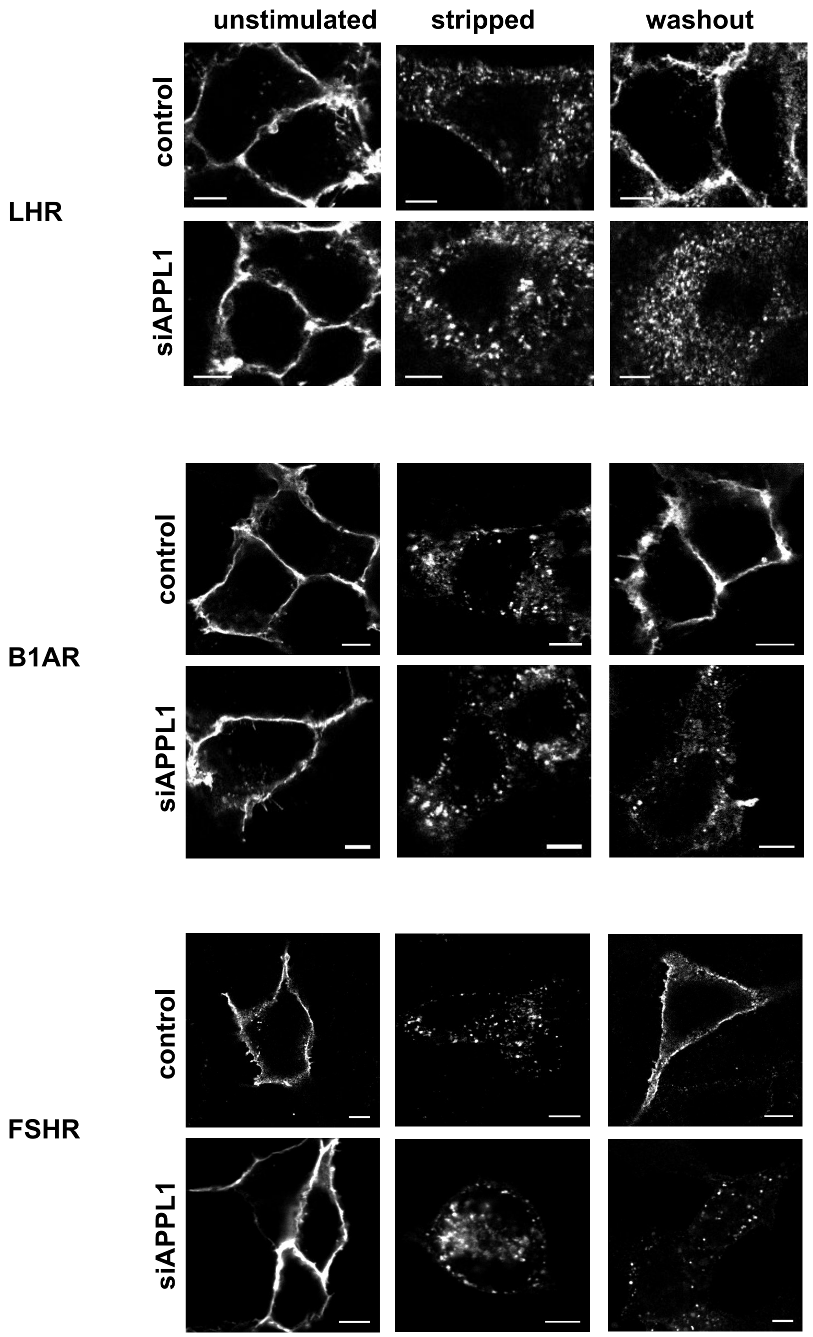

Figure S1. APPL1 is essential for GPCR recycling from VEEs, Related to figure 1D-E. Ligand-induced internalization and recycling following APPL1 siRNA-mediated knockdown were analyzed by confocal microscopy. HEK 293 cells expressing FLAG-LHR, -B1AR or -FSHR were treated with AlexaFluor555 conjugated-FLAG antibody prior to treatment with LH (10 nM), isoproterenol (ISO, 10 μM) or FSH (10 nM) for 20 min respectively. Surface bound FLAG antibody was removed by PBS/EDTA wash and incubated in medium for 1 h to allow receptor recycling. n=15 cells per condition collected across 3 independent experiments. Scale bar= 5 μm.

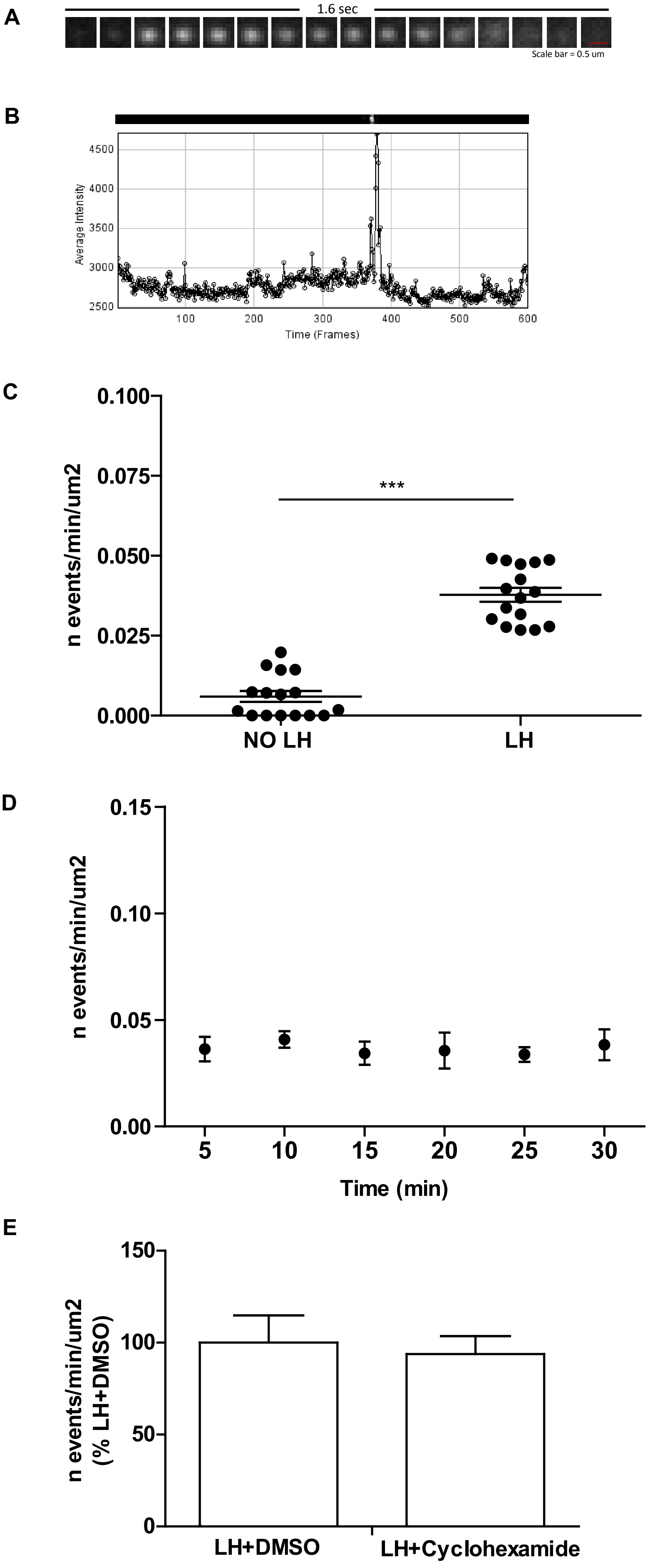

Figure S2. Characterization of SEP-LHR recycling events via TIR-FM, Related to Figure 1E. (A) Representative TIR-FM image of a SEP-LHR recycling event following stimulation with LH (10 nM). (B) Maximum intensity analysis from Fig. S2A. (C) Number of recycling events observed in HEK 293 cells stably expressing SEP-LHR ± LH (10 nM). n=16 cells per condition. ***p<0.001. (D) Number of recycling events over time observed in HEK 293 cells stably expressing SEP-LHR, stimulated with LH (10 nM). n=3-5 cells/time point. (E) Number of recycling events over time observed in HEK 293 cells stably expressing SEP-LHR, ± cyclohexamide pre-tratement (10 mg/mL 1.5 h, conditions reported to inhibit *de novo* receptor synthesis (Bremnes et al., 2000; Gicquiaux et al., 2002)) prior to LH stimulation (10 nM). n=6 cells per condition.

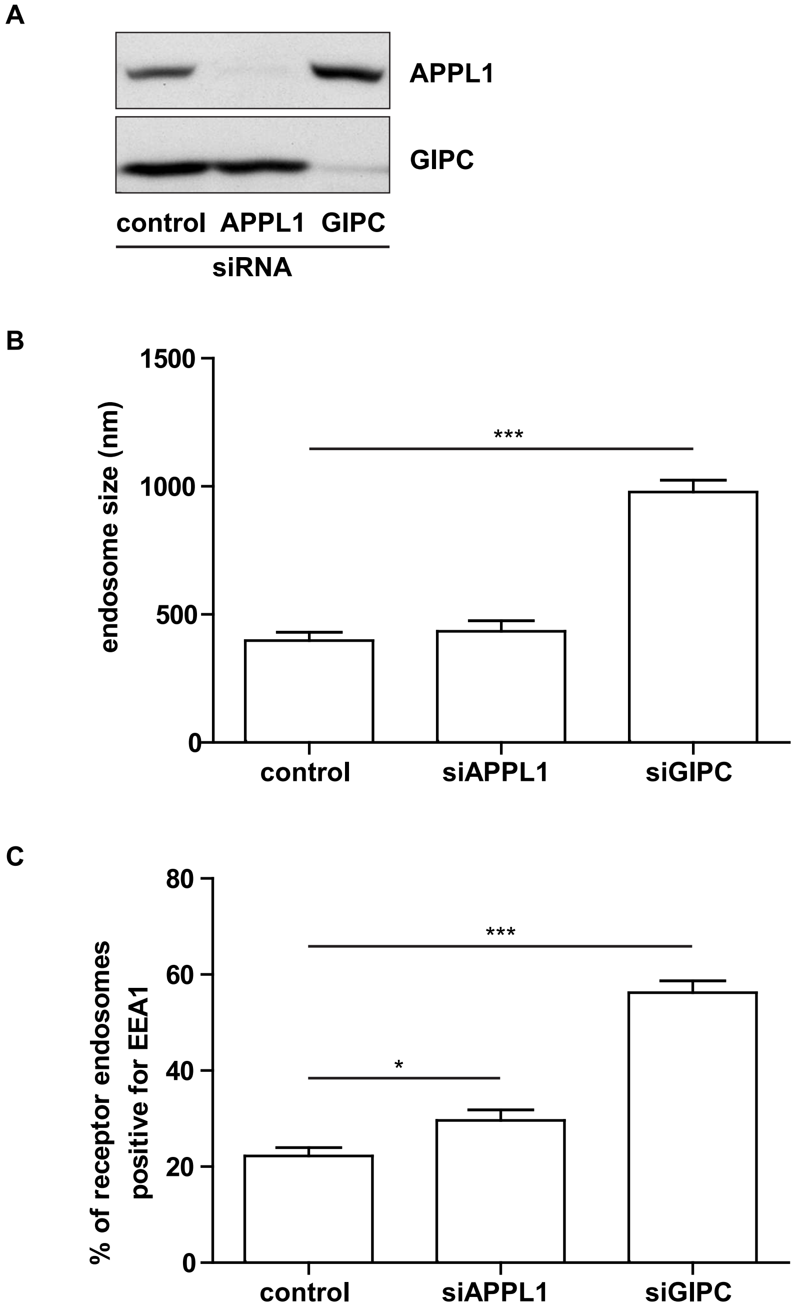

Figure S3. LHR is not re-routed to EEs following APPL1 depletion, Related to Figure 1E. (A) Representative western blot of total cellular levels of APPL1 or GIPC from lysates collected from HEK 293 cells expressing FLAG-LHR following either scamble (control), APPL1 (siAPPL1) or GIPC (siGIPC) siRNA-mediate knockdown. (B) Size of endosomes containing internalized FLAG-LHR in cells transfected with either scamble (control), APPL1 (siAPPL1) or GIPC (siGIPC) siRNA. Cells were imaged live by confocal microscopy following 10-15 min of LH (10 nM) treatment. Endosome size was quantified using Leica LASAF software, n=6 cells per condition. ***p<0.001. (C) Quantification of LHR endosomes positive for EEA1 in HEK 293 cells expressing FLAG-LHR following either scamble (control), APPL1 (siAPPL1) or GIPC (siGIPC) siRNA-mediate knockdown. Cells were labelled with AlexaFluor488-conjugated antibody, stimulated with LH (10 nM, 10 min), fixed, permeabilized, stained with EEA1 antibody and imaged via confocal microscopy. n=27 cells per condition. *p<0.05, ***p<0.001.

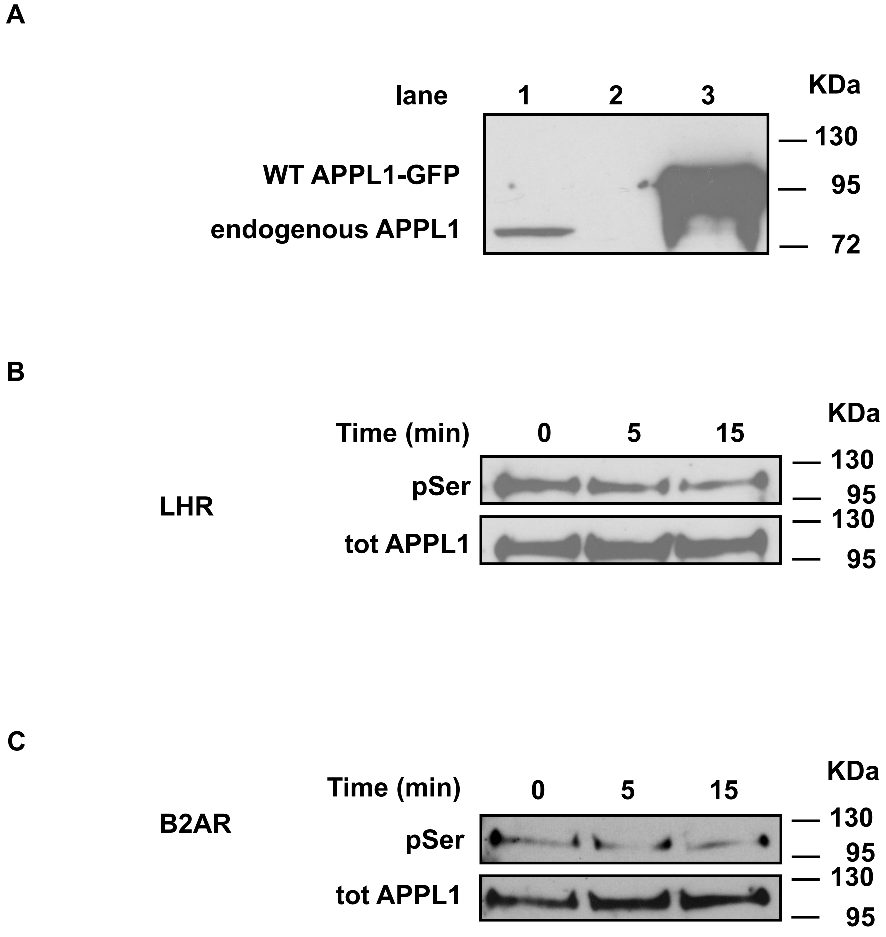

Figure S4. GFP-APPL1 is specifically immunoprecipitated via a GFP Nanobody and not phosphorylated by B2AR or direct activation of PKA, Related to Figure 3. (A) HEK 293 cells stably expressing FLAG-LHR were transfected with or without GFP-APPL1. After collection of lysates, GFP-APPL1 was immunoprecipitated using GFP nanobody (Nano-Trap) and APPL1 levels were determined with an anti-APPL1 antibody. Lane 1, untransfected cell lysates; Lane 2, untransfected cell lysates immunoprecipitated with GFP nanobody; Lane 3, cell transfected with GFP-APPL1, lysed and immunoprecipitated with GFP nanobody. (B-C) HEK 293 cells stably expressing FLAG-LHR (B) or FLAG-B2AR (C) and transfected with GFP-APPL1 were stimulated with either 8-Br-cAMP (0.5 mM) or isoproterenol (10 μM), respectively, for 0, 5 or 15 min. After collection of lysates, GFP-APPL1 was immunoprecipitated using GFP nanobody (GFP -Trap) and and both phosphoserine and APPL1 levels were determined by w estern blot. Representative immunoblots of phosphoserine (pSer) and total APPL1 (tot APPL1).

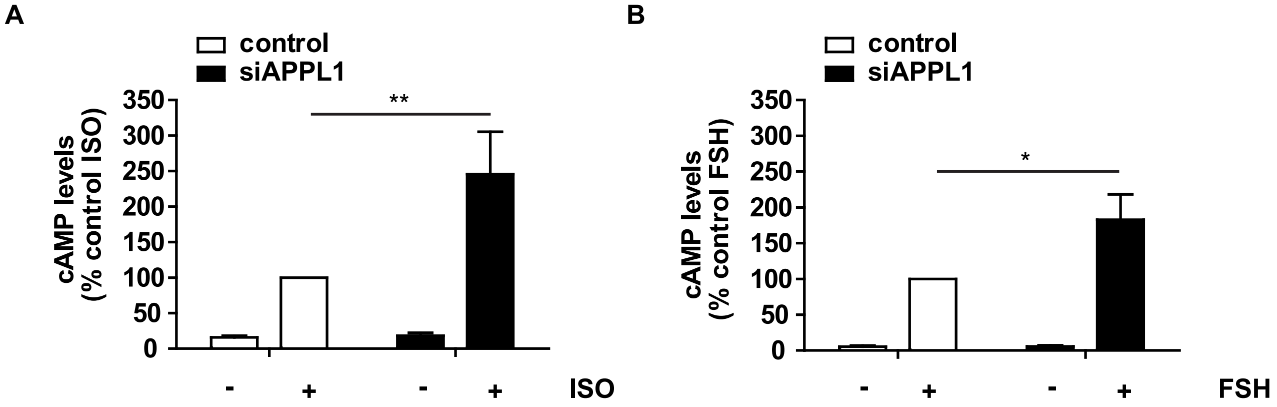

Figure S5. APPL1 negatively regulates cAMP signaling from distinct VEE-localized GPCRs, Related to Figu re 4A-B. Intracellular levels of cAMP were measured in HEK 293 cells expressing either FLAG-B1AR (A) or FLAG-FSHR (B) following transfection with either scrable siRNA (control) or APPL1 siRNA (siAPPL1). Cells were stimulated with and without isoproterenol (ISO, 10 μM, 5 min) or FSH (10 nM, 5 min). n=4 for (A) and n=3 for (B). *p<0.05, **p<0.01.

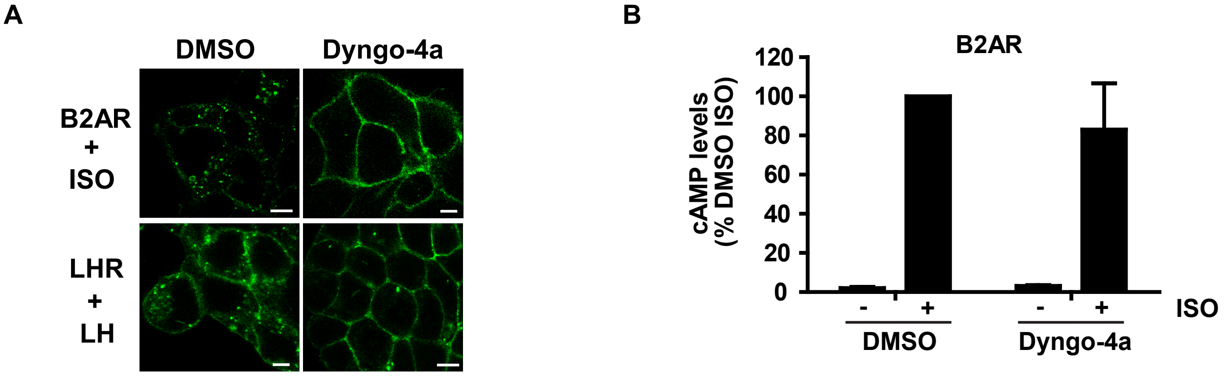

Figure S6. Dyngo-4a blocks internalization of both LHR and B2AR, but does not alter cAMP levels produced by B2AR, Related to Figure 6A. (A) HEK 293 cells expressing either FLAG-B2AR or FLAG-LHR were labelled with AlexaFluor488 conjugated FLAG-antibody, pre-treated with either DMSO or Dyngo-4a (30 µM, 45 min) and stimulated with either isoproterenol (ISO, 10 µM, 5 min) or LH (10 nM, 5 min). Ligand induced internalization of receptor was analyzed by confocal microscopy. Scale bar=10 µm. (B) Intracellular levels of cAMP measured in cells expressing FLAG-B2AR ± stimulation with isoproterenol (ISO, 10 µM, 5 min) and pre-treatment with either DMSO or Dyngo-4a (30 µM, 45 min). n=5 independent experiments.

Figure	PCC	SE
2B (control)	0.78720	0.035278
2B (KT)	0.78239	0.025581
2D (WT)	0.724423	0.018096
2D (S/A)	0.743141	0.0163
2D (S/D)	0.752436	0.02117

Table S1. Pearson's correlation coefficient for APPL1-LHR co-localization, Related to Figure 2. PCC was calculated for cells analysed in Figure 2B and D selecting at least 3 ROIs per cells, n=15 cells per condition.

Supplemental References

Bremnes, T., Paasche, J. D., Mehlum, A., Sandberg, C., Bremnes, B. & Attramadal, H. 2000. Regulation and intracellular trafficking pathways of the endothelin receptors. *J Biol Chem,* 275**,** 17596-604

Gicquiaux, H., Lecat, S., Gaire, M., Dieterlen, A., Mely, Y., Takeda, K., Bucher, B. & Galzi, J. L. 2002. Rapid internalization and recycling of the human neuropeptide Y Y(1) receptor. *J Biol Chem,* 277**,** 6645-55
